# Population study evaluating fracture risk among patients with chronic osteomyelitis

**DOI:** 10.1371/journal.pone.0189743

**Published:** 2017-12-21

**Authors:** Chyi Lo, Fung-Chang Sung, Chih-Hsin Mou, Tzu-Chieh Lin, Chun-Huang Tseng, Ya-Ling Tzeng

**Affiliations:** 1 School of Nursing, College of Health Care, China Medical University, Taichung, Taiwan; 2 Department of Nursing, China Medical University Hospital, Taichung, Taiwan; 3 Management Office for Health Data, China Medical University Hospital, Taichung, Taiwan; 4 Department of Health Services Administration, China Medical University, Taichung, Taiwan; 5 Division of Traumatology, Emergency Department, Taichung Veterans General Hospital, Taichung, Taiwan; 6 Department of Public Health, China Medical University, Taichung, Taiwan; 7 Department of Neurology, China Medical University Hospital, Taichung, Taiwan; Kanazawa University, JAPAN

## Abstract

**Background:**

Studies investigating the fracture risk in patients with chronic osteomyelitis (COM) limited to case reports. This study evaluated the association between COM and subsequent fracture risk using population-based data.

**Methods:**

A subset claims data of the Taiwan National Health Insurance was used to identify 7,147 patients with COM newly diagnosed in 1999–2005 without fracture history and 28,588 general population controls, frequency matched by sex, age and diagnosis date. The incident fractures was measured by the end of 2013.

**Results:**

The incidence density of fracture was 1.94-fold greater in the COM cohort than in controls (21.5 vs. 11.1 per 1000 person-years), with the adjusted hazard ratio (HR) of 1.81 (95% CI: 1.67–1.95) for COM patients compared to controls after controlling for sex, age, and comorbidities of diabetes, osteoporosis, depression and end-stage renal disease in Cox proportional hazards regression. The fracture risk increased with age and women were at greater risk than men. The fracture incidence increased substantially in those with osteoporosis, 40.2 per 1000 person-years in COM patients. Site specific analysis showed a higher portion of incident fractures for lower limbs, 52.7% in COM cohort and 46.3% in controls.

**Conclusion:**

Findings in this 15-year follow-up observation support our hypothesis that patients with COM are at an elevated risk of subsequent fracture. COM patients and the elderly deserve adequate consultation and awareness for fracture prevention.

## Introduction

Individuals with bone disorders are at an elevated risk for fracture, which may lead to complications. Fractures or shatters of bone are usually associated with falls and accidents, or other excessive force applied to the bone. Old age, low bone mineral density, and previous fracture are strong risk factors for fractures at almost skeletal sites [1]. The elderly with low density bone weakened from calcium depletion are at a particularly higher risk of fracture [2,3]. The younger with osteogenesis imperfecta due to brittle bone are also at an increased risk of bone fractures with little trauma [4].

Osteomyelitis is often an acute infection of short-term, but it may progress into a chronic inflammation phase [5]. Chronic osteomyelitis (COM) is also a bone disorder with inflammation of the bone, resulting in the destruction of bone formation and fistulous tracts [6,7]. COM develops progressively over a period of weeks, months, or even years after the onset of infection [8,9]. Plain radiography is an appreciated method to differentiate COM from other bone disorders, and to monitor treatment progress [9,10]. Osteomyelitis is not only a common complication in open or closed fracture, it can also devastatingly lead to loss of bone substance and vascular and soft tissue injuries [8,11,12]. COM may lead to other avascular necrosis of bone and dead bone because of the formation of sequestrum [6,13]. Consequently, patients with COM may further lead to other bone disorders or cause pathological fracture [14–16]. An amputation may be required for the severe case [17–19]. In an infectious disease clinic study with 75 patients of diabetic foot infection, Yapici et al. found that these patients were at higher risk of developing osteomyelitis (53 cases or 70.7%) and amputation (25 cases or 33.3%). Findings from these studies suggest a potential risk of fractures for patients with COM [18].

Studies on the association between osteomyelitis and the risk of fractures limit to case reports [14,20–22]. A 13-year old Ugandan boy with chronic osteomyelitis because of *Mycotic aneurysm* infection experienced a pathological fracture of the femur [20]. Lin et al. (2010) reported a case of pathological fracture of the right distal radius for a 79-year old male patient with *Enterobacter aerogenes* osteomyelitis [21]. Döring et al. (2016) found recently that the displaced neck of femur fracture for an old patient was associated with chronic osteomyelitis of the ipsilateral foot. No other types of study have been conducted on the fracture risk for patients with COM. We hypothesized that a population-based study might be able to evaluate this relationship. We, therefore, used the claims data of National Health Insurance (NHI) of Taiwan to examine the risk of fracture for patients with COM.

## Methods and materials

### Data source

The NHI program of Taiwan is a single-payer system integrated from 13 public insurance systems in 1995, with nearly 99% of population covered by 1999 [23]. We obtained from the Taiwan National Health Research Institutes (NHRI) a subset of longitudinal electronic data with medical claims of insured population for the 1996–2013 period. The diagnoses of disease consisted of one primary code and four second codes using the International Classification of Diseases, 9^th^ Revision, Clinical Modification (ICD-9-CM) for each claim.

### Study patients

From the NHI database, we identified 14,115 patients with osteomyelitis, presenting for more than a month, newly diagnosed from 1999 to 2005. We excluded 6,968 patients who had a fracture history (ICD-9-CM code 800–829) by the entry date. The remaining 7,147 patients (ICD-9-CM code 730.1) were included in the chronic osteomyelitis (COM) cohort. The first date with COM diagnosed was designated as the date for entering the study. For each COM patient, four persons without the history of osteomyelitis and fracture were randomly selected into the non-COM cohort as controls, frequency matched by sex, age (every 5 years), and entry-year and entry-month. We assessed the incident fracture in each study cohort. Each study subject was followed from the entry date until the date with fracture diagnosed, or censored because of death, withdrawal from the insurance program or the end of 2013. The potential comorbidities associating with the COM and fracture in this study included diabetes (ICD-9-CM code 250), osteoporosis (ICD-9-CM code 733.0), depression (ICD-9-CM code 296.2, 296.3, 300.4 and 311) and end-stage renal disease (ICD-9-CM code 585 from the registry of catastrophic illness patients).

### Statistical analysis

All statistical analyses were performed using SAS version 9.2 (SAS Institute Inc, Cary, NC) and the significant level was set 0.05 at two-tailed test. Baseline distributions of sex, age (< 45, 45–59, 60–74 and ≥ 75 years) and comorbidities were compared between COM and non-COM cohorts and examined using Chi-square. The cumulative fracture free rates of both cohorts were presented with plot using Kaplan-Meier method and the difference between two plots was examined using log-rank test. Incidences of fracture were counted by dividing the number of incident fracture by follow-up person-years. We used Cox proportional hazards regression to estimate the hazard ratios (HRs) and 95% confidence intervals (CIs) of fracture. Multivariable Cox model was used to calculate adjusted HRs after controlling sex, age, diabetes, osteoporosis, depression and end-stage renal disease. Sex- and age-specific incidence rates were calculated by age and sex for both cohorts; HRs of incident fracture were calculated using 20–44 year women without COM as the reference. We also assessed the fracture by site, including head (ICD-9-CM code 800–804), body (ICD-9-CM code 805–809), upper arm (ICD-9-CM code 810–819) and low limb (ICD-9-CM code 820–829). The further data analysis evaluated joint effects on fracture associating with diabetes, osteoporosis, depression and end-stage renal disease, comparing with individuals in the control cohort without these comorbidities.

### Ethics and consent

All identification numbers of insured population had been encrypted and replaced with surrogate identifiers. Therefore, this study requires no informed consents of the study population with the approval from the Research Ethics Committee at China Medical University and Hospital (CMUH-104-REC2-115).

## Results

Both the COM cohort (N = 7,147) and the control cohort (N = 28,588) were similar in sex and age distributions (Table 1). Men were more prone to osteomyelitis. Half of study population were 60 years old or older. The chronic osteomyelitis cohort was more prevalent with comorbidities of diabetes, osteoporosis, depression and end-stage renal disease.

**Table 1:** Comparison in demographic status and comorbidity between cohorts with and without chronic osteomyelitis.

	Non- Osteomyelitis N = 28,588		Chronic Osteomyelitis N = 7,147		
Variable	N	%	n	%	p-value
Gender					0.99
Women	10584	37.0	2646	37.0	
Men	18004	63.0	4501	63.0	
Age, years					0.99
< 45	6812	23.8	1703	23.8	
45–59	7488	26.2	1872	26.2	
60–74	9624	33.7	2406	33.7	
≥ 75	4664	16.3	1166	16.3	
Comorbidity					
Diabetes mellitus	1548	5.41	2328	32.6	<0.0001
Osteoporosis	157	0.55	256	3.58	<0.0001
Depression	82	0.29	86	1.20	<0.0001
End-stage renal disease	109	0.38	224	3.13	<0.0001

Chi-square test

**Fig 1:**
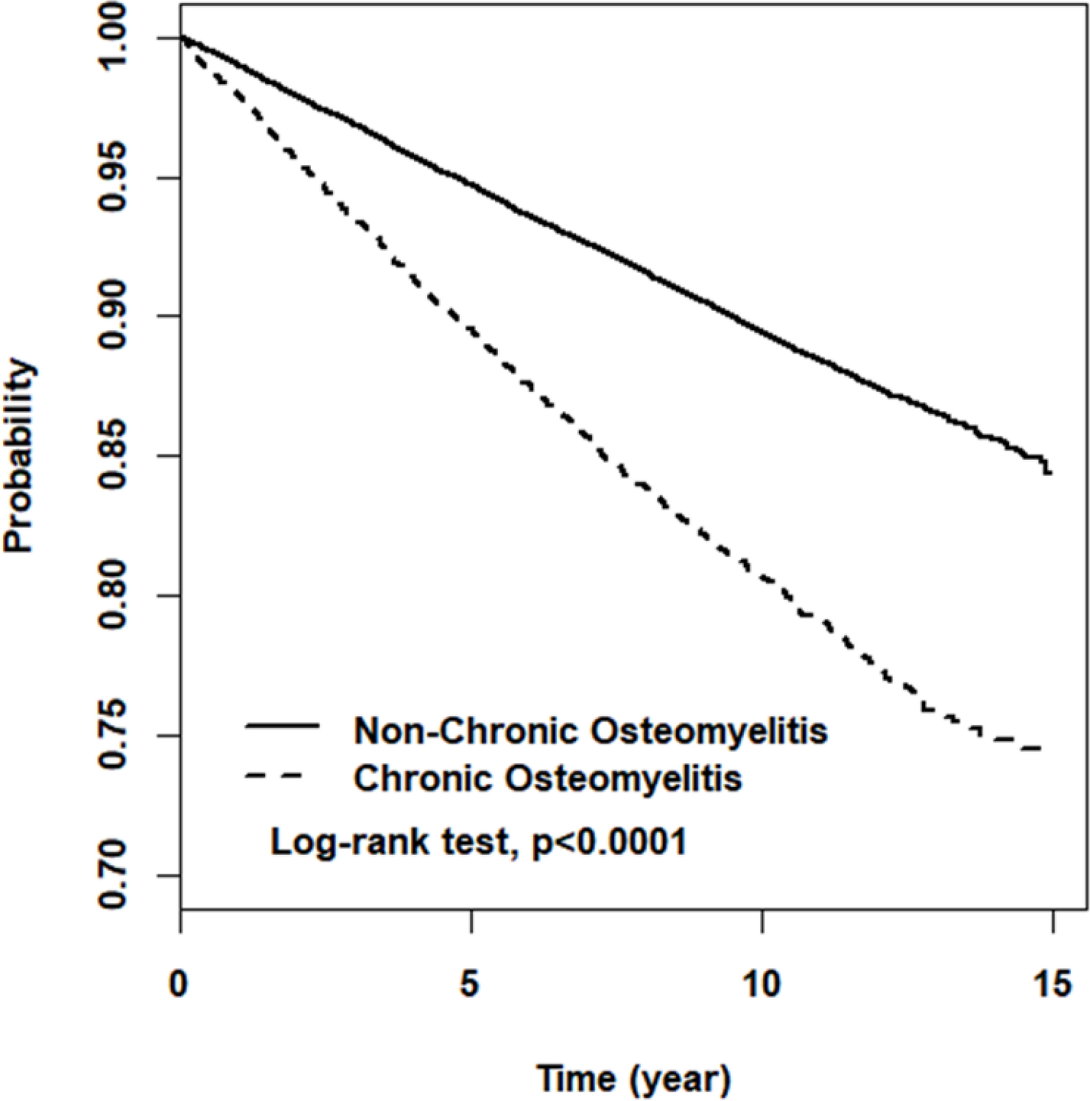
Fracture-free rate between chronic osteomyelitis and non-chronic osteomyelitis group.

By the end of follow-up period, 1086 and 3004 cases of fracture in COM and control cohorts were identified after 15-year of follow-up, respectively. The proportional fracture incidence was 9.89% higher in COM patients than in controls (Log-rank test *p* <0.001) (Fig 1). Compared to control cohort, the incidence density of fracture in COM cohort was 1.94-fold greater than controls (21.5 vs. 11.1 per 1000 person-years) with an adjusted HR of 1.81-fold (95% CI 1.67–1.95) after controlling for covariates (Table 2). The incident fracture increased with age in both cohorts, with the age associated fracture risk stronger for women than for men (Table 3). The fracture incidence was the greatest for women with COM of 75 years and older, 14.6-fold greater than 20–44 years women without COM, with an adjusted HR of 14.6 (95% CI 10.4–20.4).

**Table 2:** Incidence and Cox method estimated hazard ratio of fracture by sex and age.

	Chronic Osteomyelitis						Compared to Non-Osteomyelitis			
	No			Yes			Crude		Adjusted	
Variable	Case	P-Y	IR	Case	P-Y	IR	HR (95% CI)		HR (95% CI)	
Overall	3004	270187	11.1	1086	50527	21.5	1.94	(1.81–2.07)	1.81	(1.67–1.95)
Gender										
Female	1442	99822	14.5	459	19365	23.7	1.65	(1.49–1.84)	1.52	(1.36–1.71)
Male	1562	170365	9.2	627	31162	20.1	2.18	(1.99–2.39)	2.08	(1.88–2.30)
Age, years										
< 45	356	73936	4.8	211	16086	12.1	2.70	(2.28–3.20)	2.48	(2.08–2.97)
45–59	573	78292	7.3	294	14418	20.4	2.81	(2.44–3.23)	2.21	(1.87–2.60)
60–74	1260	88053	14.3	402	15147	26.5	1.92	(1.71–2.15)	1.58	(1.40–1.79)
≥ 75	815	29905	27.3	179	4877	36.7	1.37	(1.16–1.61)	1.29	(1.09–1.53)

P-Y, person-years; IR, incidence; HR, hazard ratio; CI, confidence interval. Incidence, per 1,000 person-years.

Adjusted HR: mutual adjusted for age, gender, diabetes, osteoporosis, depression and end-stage renal disease in Cox proportional hazards regression.

**Table 3:** Incident fracture estimated by age and sex in cohorts with and without chronic osteomyelitis.

	Female								Male							
	Non-Chronic Osteomyelitis				Chronic Osteomyelitis				Non-Chronic Osteomyelitis				Chronic Osteomyelitis			
Age, years	Case	IR	HR	(95% CI)	Case	IR	HR	(95% CI)	Case	IR	HR	(95% CI)	Case	IR	HR	(95% CI)
< 45	53	2.5	1.00	(reference)	32	6.7	2.57	(1.65–3.98)	303	5.7	2.27	(1.70–3.05)	179	15.8	6.05	(4.45–8.22)
45–59	226	8.7	3.43	(2.54–4.63)	114	22.2	7.74	(5.58–10.7)	347	6.6	2.63	(1.97–3.51)	180	19.4	6.80	(4.99–9.26)
60–74	707	17.7	6.87	(5.19–9.08)	214	29.6	9.90	(7.30–13.4)	553	11.5	4.51	(3.40–5.98)	188	23.7	8.26	(6.07–11.2)
≥ 75	456	35.4	13.6	(10.2–18.1)	99	44.1	14.6	(10.4–20.4)	359	21.1	8.36	(6.26–11.2)	80	30.4	10.9	(7.67–15.4)

IR, incidence rate per 1,000 person-years; HR, hazard ratio; CI, confidence interval.

Adjusted HR: adjusted for diabetes, osteoporosis, depression and end-stage renal disease in Cox proportional hazards regression.

COM patients with comorbidities of diabetes, depression and end-stage renal disease were at greater risk to develop fracture than those in the non-COM cohort (Table 4). The fracture incidence increased further to 40.2 per 1000 person-years for COM patients with osteoporosis, 2.23-fold greater than those with only COM. COM patients with more than two comorbidities had an incidence of 33.1 per 1000 person-years, with an aHR of 2.67 (95% CI 1.90–3.74) compared with controls without comorbidity.

**Table 4:** Joint effect of fracture risk in chronic osteomyelitis and comorbidity.

	Non-Chronic Osteomyelitis				Chronic Osteomyelitis			
Variable	Case	IR	aHR	(95% CI)	Case	IR	aHR	(95% CI)
None	2711	10.5	1.00		657	18.0	1.93	(1.77–2.10)
With only diabetes	232	25.0	1.67	(1.46–1.92)	342	29.9	2.53	(2.26–2.83)
With only osteoporosis	36	46.2	2.52	(1.81–3.51)	40	40.2	2.32	(1.70–3.18)
With only depression	8	16.9	1.25	(0.63–2.51)	8	26.2	2.59	(1.29–5.19)
With only end-stage renal disease	4	9.9	0.83	(0.31–2.21)	5	28.0	2.43	(1.01–5.85)
With more than two comorbidities	13	32.6	1.96	(1.14–3.39)	34	33.1	2.67	(1.90–3.74)

P-Y, person-years; IR, incidence rate per 1,000 person-years; HR, hazard ratio; CI, confidence interval.

aHR, adjusted HR: adjusted for age and gender.

Table 5 shows the fracture occurrence by site was most often to lower limbs (11.3 vs. 5.15 per 1000 person-years in COM patients and controls, respectively, followed by the body or upper arms and the lowest for head. The incidence of head fracture was greater in men than in women, while women had greater incident fractures of body, upper arms and lower limbs.

**Table 5:** Incidence and hazard ratio of fracture by site and sex.

	Chronic Osteomyelitis				Compared to Non-Osteomyelitis	
	No		Yes			
Site (ICD-9-CM)	Case	IR	Case	IR	Adjusted HR (95% CI)	
All						
Head (800–804)	161	0.60	50	0.99	1.65	(1.17–2.32)
Body (805–809)	644	2.38	230	4.55	1.84	(1.54–2.14)
Upper arms (810–819)	808	2.99	234	4.63	1.58	(1.35–1.84)
Low limbs (820–829)	1391	5.15	572	11.3	1.96	(1.76–2.18)
Female						
Head (800–804)	31	0.31	7	0.36	1.20	(0.50–2.86)
Body (805–809)	298	2.99	97	5.01	1.61	(1.26–2.07)
Upper arms (810– 819)	411	4.12	110	5.68	1.49	(1.19–1.87)
Low limbs (820–829)	702	7.03	245	12.6	1.54	(1.31–1.80)
Male						
Head (800–804)	130	0.76	43	1.38	1.76	(1.21–2.55)
Body (805–809)	346	2.03	133	4.27	1.99	(1.60–2.48)
Upper arms (810–819)	397	2.33	124	3.98	1.64	(1.32–2.03)
Low limbs (820–829)	689	4.04	327	10.5	2.46	(2.13–2.84)

P-Y, person-years; IR, incidence rate per 1,000 person-years; HR, hazard ratio; CI, confidence interval.

Adjusted HR: mutual adjusted for age, gender, diabetes, osteoporosis, depression and end-stage renal disease in Cox proportional hazards regression.

## Discussion

Studies have linked fracture risk to old age, female, diabetes, osteoporosis and depression [24–34]. Osteoporosis has long been recognized as the major factor leading to fracture. The present study found COM is also a risk factor of fracture. Our findings confirmed that patients with COM had a near 2-fold greater risk of fracture than those without COM. The fracture risk for COM patients increased further for those with the comorbidity of diabetes, osteoporosis, depression or end-stage renal disease. The greatest increase was to 40.2 per 1000 person-year in those with osteoporosis.

Osteoporosis is the well-known factor associated with an increased risk of fracture because of weak and brittle bones [35–37]. Women are generally at a higher risk than men because women have a lower bone density with a higher prevalence of osteoporosis after menopause that dispose to fracture [28,35,38].

Old age is a known key risk factor of fracture. This study showed that younger women without COM had the lowest incidence of fracture, but the age related risk is in a greater increase in women than in men in both cohorts. The fracture incidence was 6.6-fold greater for ≥75-year women than for <45-year women in the COM cohort (44.1 vs. 6.7 per 1000 person-years), while the corresponding incidence ratio was 1.9 folds (30.4 vs. 15.8 per 1000 person-years) for men. It is likely the mechanism of a greater fracture risk for women with COM could be related to a higher prevalence of osteoporosis in women, particularly in older women.

Patients with COM may suffer from avascular necrosis of bone and formation of dead bone [3,13,39]. We suspect that these condition may interact with osteoporosis to further weaken the bone in older women. The fracture risk is greater for women than for men when excessive force applied to the bone. Further data analysis showed, among 1086 fracture cases in the COM cohort, 463 cases were associated with falls, 353 cases were associated with accidents and 270 cases were unspecified (Table 6). The fracture incidence due to falls was 1.8-fold greater for women than for men (12.65 vs. 7.00 per 1000 person-years). On the other hand, the risk associating with accidents was 37% lower for women than for men (5.11 vs. 8.15 per 1000 person-years). This contrast demonstrates that the excessive force applied to the bone is greater from accidents than from falls.

**Table 6:** Incidence and hazard ratio of fracture associated with fall and traffic accident by sex.

	Chronic Osteomyelitis				Compared to Non-Osteomyelitis	
	No		Yes			
	Case	IR	Case	IR	Adjusted HR (95% CI)	
All						
Fall	1281	4.74	463	9.16	1.80	(1.60–2.02)
Accident	993	3.68	353	6.99	1.82	(1.59–2.07)
Other	730	2.70	270	5.34	1.83	(1.57–2.13)
Female						
Fall	749	7.50	245	12.6	1.50	(1.28–1.76)
Accident	333	3.34	99	5.11	1.47	(1.16–1.88)
Other	360	3.61	115	5.94	1.66	(1.32–2.08)
Male						
Fall	532	3.12	218	7.00	2.29	(1.93–2.71)
Accident	660	3.87	254	8.15	1.99	(1.70–2.33)
Other	370	2.17	155	4.97	1.97	(1.60–2.43)

P-Y, person-years; IR, incidence rate per 1,000 person-years; HR, hazard ratio; CI, confidence interval.

Adjusted HR: mutual adjusted for age, gender, diabetes, osteoporosis, depression and end-stage renal disease in Cox proportional hazards regression analysis.

Men are more likely than women to be involved in accidents. This hypothesis can also be explained in the site specific fracture data: men are more likely to have head fracture because of accidents. The results of age- and sex-specific analysis showed that the hazard of fracture for the young COM patients was much greater for men than for women (aHR: 6.05 vs. 2.57). It is possible that young men are more likely than young women to be involved in accidents and experienced the fracture.

### Strengths and limitations

The present study took the advantage of a large population data to examine the hypothesis on the relationship between COM and fracture risk. The 15-year observation enhances our ability to identify fracture development subsequent to the chronic inflammation of the bone. The long-term follow-up of natural health history for the insured population reduces the potential surveillance bias. The large study cohorts enabled us to gain adequate power to stratify data into subgroups to ascertain the impact of COM on the risk of incident fractures by sex, age and comorbidity. However, there were several limitations for comment. COM is a disorder generally secondary to an infection after trauma or surgery. This study excluded patients with the history of fracture, thereby detecting a newly developed fracture. Previous case reports have associated COM with pathologic fractures [14,20,21]. We suspected that some cases among the 270 fracture cases unspecified as accidents or falls in the COM cohort were pathologic related. In addition, COM remains difficult to treat completely. However, information on laboratory tests were unavailable in the claims data. We were unable to distinguish whether COM patients were cured or not, and unable to identify the pathologic related fracture. Further study should be warranted to identify the COM related pathologic fracture and non-pathologic fracture, and to investigate the clinical effectiveness of therapy for the disease. Furthermore, information on lifestyle and health behavior, such as calcium vitamin consumption, smoking and drinking and exercise, was also unavailable for adjustment in the data analysis[40–42]. This bias may become non-distinguishable due to using a large population database with a long observation period.

## Conclusions

The present study reveals an important role of COM in association with the risk of fracture. Patients with COM are at an elevated risk of developing fracture, regardless of the presence or absence of the comorbidities. The fracture risk for patients with COM could be greatly increased because of comorbidities, particularly for the elderly and those with osteoporosis. The site with the highest incidence of fracture was lower limbs. Both fall and accident are the causes leading to fracture for COM patients. These findings could be used to prompt clinical alerts. Clinicians should provide COM patients with timely consultation on fracture prevention, particularly of knowledge in fall and accident prevention for the elderly patients with osteoporosis and other comorbidities.
